# Future research trends in understanding the mechanisms underlying allergic diseases for improved patient care

**DOI:** 10.1111/all.13851

**Published:** 2019-06-04

**Authors:** Heimo Breiteneder, Zuzana Diamant, Thomas Eiwegger, Wytske J. Fokkens, Claudia Traidl‐Hoffmann, Kari Nadeau, Robyn E. O’Hehir, Liam O’Mahony, Oliver Pfaar, Maria J. Torres, De Yun Wang, Luo Zhang, Cezmi A. Akdis

**Affiliations:** ^1^ Institute of Pathophysiology and Allergy Research Medical University of Vienna Vienna Austria; ^2^ Department of Respiratory Medicine & Allergology, Institute for Clinical Science, Skane University Hospital Lund University Lund Sweden; ^3^ Department of Respiratory Medicine, First Faculty of Medicine Charles University and Thomayer Hospital Prague Czech Republic; ^4^ Division of Immunology and Allergy Food Allergy and Anaphylaxis Program The Department of Pediatrics The Hospital for Sick Children Toronto Ontario Canada; ^5^ Research Institute, The Hospital for Sick Children, Translational Medicine Program Toronto Ontario Canada; ^6^ Department of Immunology The University of Toronto Toronto Ontario Canada; ^7^ Department of Otorhinolaryngology Amsterdam University Medical Centres, Location AMC Amsterdam The Netherlands; ^8^ Chair and Institute of Environmental Medicine UNIKA‐T, Technical University of Munich and Helmholtz Zentrum München Augsburg Germany; ^9^ Christine Kühne Center for Allergy Research and Education Davos Switzerland; ^10^ Sean N. Parker Center for Allergy & Asthma Research Stanford University Stanford California; ^11^ Department of Respiratory Medicine, Allergy and Clinical Immunology, Central Clinical School Monash University Melbourne Victoria Australia; ^12^ Allergy, Asthma and Clinical Immunology Service Alfred Health Melbourne Victoria Australia; ^13^ Departments of Medicine and Microbiology, APC Microbiome Ireland National University of Ireland Cork Ireland; ^14^ Department of Otorhinolaryngology, Head and Neck Surgery, Section of Rhinology and Allergy University Hospital Marburg, Philipps‐Universität Marburg Marburg Germany; ^15^ Allergy Unit Regional University Hospital of Malaga IBIMA‐UMA‐ARADyAL Malaga Spain; ^16^ Department of Otolaryngology Yong Loo Lin School of Medicine National University of Singapore Singapore; ^17^ Department of Otolaryngology Head and Neck Surgery and Department of Allergy Beijing Tongren Hospital Beijing China; ^18^ Swiss Institute of Allergy and Asthma Research (SIAF), University Zurich Davos Switzerland

**Keywords:** allergy, exposome, microbiome, neuroimmune, respiratory viral infections

## Abstract

The specialties of allergy and clinical immunology have entered the era of precision medicine with the stratification of diseases into distinct disease subsets, specific diagnoses, and targeted treatment options, including biologicals and small molecules. This article reviews recent developments in research and patient care and future trends in the discipline. The section on basic mechanisms of allergic diseases summarizes the current status and defines research needs in structural biology, type 2 inflammation, immune tolerance, neuroimmune mechanisms, role of the microbiome and diet, environmental factors, and respiratory viral infections. In the section on diagnostic challenges, clinical trials, precision medicine and immune monitoring of allergic diseases, asthma, allergic and nonallergic rhinitis, and new approaches to the diagnosis and treatment of drug hypersensitivity reactions are discussed in further detail. In the third section, unmet needs and future research areas for the treatment of allergic diseases are highlighted with topics on food allergy, biologics, small molecules, and novel therapeutic concepts in allergen‐specific immunotherapy for airway disease. Unknowns and future research needs are discussed at the end of each subsection.

## 1 INTRODUCTION

The past decades have witnessed extensive progress in unraveling cellular and molecular mechanisms of immune regulation in asthma, allergic diseases, organ transplantation, autoimmune diseases, tumor biology, and chronic infections.^1^, ^2^ Consequently, a better understanding of the functions, the reciprocal regulation, and the counterbalance of subsets of immune and inflammatory cells but also structural cells—for example, epithelial and vascular cells, airway smooth muscle cells, neuroendocrine system—that interact via various intercellular messengers will indicate avenues for immune interventions and novel treatment modalities of allergic diseases and immunological disorders. It is generally expected that drug development in the next decades will show a significant shift from chemicals to biologicals. After more than 20 years without any breakthrough drug becoming available for patients, several disciplines including allergology are now experiencing extraordinary times with the recent licensing of several major biological drugs and novel allergen‐specific immunotherapy (AIT) vaccines. Several biological modifiers of the immune response targeting intracellular messengers or their receptors have been developed to date.^3^, ^4^, ^5^, ^6^, ^7^, ^8^ In addition, a number of promising small molecule drugs and vaccines are in the development pipeline.^9^, ^10^, ^11^ This new era is now calling for the development of biomarkers and pheno‐ and endotyping of diseases for customized patient care, which is termed stratified medicine, precision medicine, or personalized medicine.^4^ Distinguishing phenotypes of a complex disease covers the observable clinically relevant properties of the disease but does not show a direct relationship to disease etiology and pathophysiology. In a complex condition, such as asthma, different pathogenetic mechanisms can induce similar clinical manifestations; however, they may require different treatment approaches.^12^, ^13^ These pathophysiological mechanisms underlying disease subgroups are addressed by the term “endotype.”^12^, ^13^, ^14^ Classification of complex diseases based on the concept of endotypes provides advantages for epidemiological, genetic, and drug‐related studies. Accurate endotyping by using reliable biomarkers reflects the natural history of the disease and aims to predict the response to (targeted) treatments.^15^ Recent studies have focused on better understanding of endotypes and phenotypes of allergic diseases, asthma, allergic and chronic rhinosinusitis ± nasal polyps, chronic obstructive pulmonary disease, and on the development of biomarkers including novel interleukins and microRNAs that regulate their expression to stratify patients.^16^, ^17^, ^18^

## 2 BASIC MECHANISMS OF ALLERGIC DISEASES—KEY QUESTIONS

### 2.1 Structural and functional biology of allergens—where are we at?

Cloning of allergen cDNAs and sequencing of purified natural allergens have so far yielded 919 officially accepted allergenic proteins listed in the database of the WHO/IUIS Allergen Nomenclature Sub‐Committee (http://www.allergen.org/; accessed 11/2018). Structures of allergens determined by crystallography or NMR amount to around 100 as summarized in the Structural Database of Allergenic Proteins (http://fermi.utmb.edu/; accessed 11/2018) and by Dall'Antonia et al^19^ Structural data of allergens allow the study of cross‐reactivities between related allergens,^20^ or the design of allergens with altered IgE epitopes as vaccine candidates for AIT.^21^ The location of IgE‐binding epitopes can be determined based on allergen structures and experimental data.^22^ Various technologies exist for mapping conformational IgE epitopes.^23^ X‐ray crystallography of an allergen‐antibody complex allows the most precise identification of conformational epitopes. To date, only two structures of cocrystals of IgE and allergen are available, Bos d 5^24^ and Phl p 2,^25^ both complexed with an IgE Fab. Sequence and structural data have revealed that allergens are members of a limited number of protein families (http://www.meduniwien.ac.at/allfam/). This insight has now become mainstream knowledge and indicates that the biological functions of allergens might be linked to their allergenicity.^26^

Various explanations for the existence of the allergic immune response have been brought forward including the toxin hypothesis,^27^, ^28^ the danger theory,^29^ and the allergic host defense model.^30^ Unequivocally, these authors^27^, ^28^, ^29^, ^30^ argue that it is a common misconception to regard allergens as generally harmless environmental substances. Allergens interact with innate immune receptors (eg, TLR4,^31^ protease‐activated receptor‐2,^32^ dectin‐1^33^), disrupt the integrity of membranes (eg, phospholipase A2,^34^, ^35^ defensins^36^, ^37^), or degrade connective tissues (eg, hyaluronidases^38^). However, very few studies on why only susceptible individuals raise an allergic immune response have come forward. They indicate that genetic susceptibility is based on altered signal processing^39^ and mutations of pattern recognition receptors.^33^

How to expand the understanding of allergens and allergic sensitization?

- Provide structures of homologous allergens to study cross‐reactivity
- Provide structures of hypoallergenic variants to visualize the effects of allergen design
- Provide structures for all major allergen types
- Provide structures for allergens complexed with IgE Fabs, IgG Fabs, or single‐chain antibodies
- Provide structures of allergens with their ligands
- Perform studies on the effect of the biological function of allergens on innate immune cells
- Perform studies on signal transduction initiated by allergens in innate immune cells
- Define pattern recognition receptors, membrane constituents, or other cellular binding partners of allergens
- Define “susceptibility to allergic sensitization” at the molecular and mechanistic level

### 2.2 Mechanisms of type 2 inflammation and immune tolerance to allergens

#### 2.2.1 Type 2 immune response

**Figure 1:**
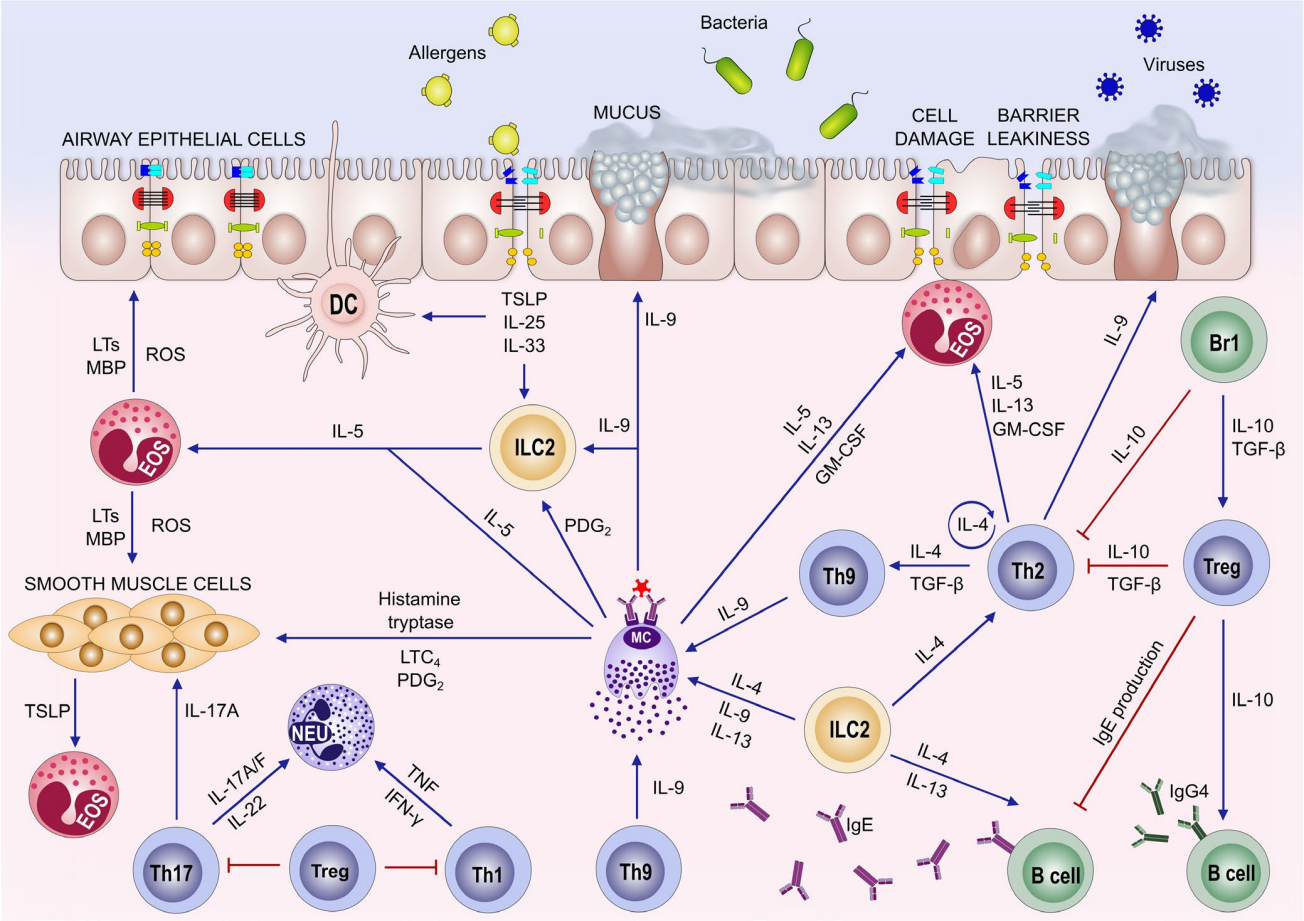
Molecular mechanisms in allergic inflammation. Epithelial leakiness and activation and their proinflammatory cytokine and chemokine (TNF‐α, IL‐13, TSLP, IL‐25, IL‐33) production induce inflammation and contribute to the Th2 response. Highly activated epithelial cells undergo apoptosis and shedding takes place. Chemokines are essential players for the recruitment of inflammatory cells followed by survival and reactivation of migrating inflammatory cells and their interaction with resident tissue cells and other inflammatory cells. Innate lymphoid cells (ILC2) play a role in T‐ and B‐cell activation and recruitment and are early providers of type 2 cytokines and T‐cell recruitment‐related chemokines. The Th2 type of an immune microenvironment is characterized by IL‐4, IL‐5, IL‐9, IL‐13, IL‐25, IL‐33 production by Th2 cells, ILCs, mast cells, and tissue cells. Eosinophilia is induced by IL‐5, IL‐25, and IL‐33. Local and systemic IgE production takes place in allergic patients with the involvement of IL‐4 and IL‐13. Other effector T‐cell subsets, such as Th9, Th17, and Th22 cells, also play partial roles in inflammation, mucus production, and tissue healing. Smooth muscle, myofibroblast activation, and bronchial hyperreactivity are related to IL‐4, IL‐9, IL‐13, IL‐25, and IL‐33. Several chemokines, and arachidonic acid pathway molecules and other small molecules play roles in the inflammatory cell recruitment and further augmentation of the inflammatory cascades. Treg and Breg cells play a role on control of inflammation and extensive cellular activation by using IL‐10 and TGF‐β as well as many other suppressive mechanisms

Since the discovery of T‐helper (Th) subsets, it was demonstrated in the last three decades that almost all immune cells display functional subsets characterized by distinct signature cytokines and surface receptors. Generally, it is considered that a type 2 immune response is the main player in the pathogenesis of eosinophilic asthma, allergic rhinitis, chronic rhinosinusitis with nasal polyps, eosinophilic esophagitis, and extrinsic atopic dermatitis.^40^ The type 2 immune response is an immune response to environmental noninfectious proteins and helminths, and involves Th2 cells, type 2 B cells, group 2 innate lymphoid cells, type 2 macrophages, a small fraction of IL‐4–secreting NK cells, IL‐4–secreting NK‐T cells, basophils, eosinophils, and mast cells.^41^, ^42^ From a complex network of cytokines, IL‐4, IL‐5, IL‐9, IL‐13, and IL‐31 are mainly secreted from immune system cells and IL‐25, IL‐33, and TSLP from tissue cells, particularly epithelial cells.^43^, ^44^ (Figure 1) GATA3 is the key transcription factor for the induction of this response.^45^ Both the innate and the adaptive immune response contribute to type 2 immune response. Among these cytokines, IL‐4 and IL‐13 play roles in production of allergen‐specific IgE, IL‐5 in eosinophilia,^46^, ^47^ IL‐9 and IL‐13 in mucus production, IL‐4 and IL‐13 in tissue migration of Th2 cells and eosinophils, and IL‐4 and IL‐13 in regulation of tight junctions and epithelial barrier integrity.^48^ Type 1, type 17, type 22, and immune regulatory responses, and nonallergic mechanisms such as environmental factors, psycho‐social stress, activation of metabolic pathways, resident cells in the remodeled phenotype, or epithelial barrier dysfunction further modulate the profile of type 2–driven inflammation. In addition, type 2–driven inflammation is characterized by a high cellular plasticity that enables the cells to adapt to a specific inflammatory milieu. Several subendotypes might exist within the type 2 immune response complex endotype such as the IL‐5‐high, IL‐13‐high, or IgE‐high endotype, and their dominance differs between allergic diseases (Figure 1). Omalizumab targeting IgE, mepolizumab, reslizumab targeting IL‐5, benralizumab targeting the IL‐5 receptor, and dupilumab targeting the IL‐4 and IL‐13 common receptor alpha chain are some of the biologicals currently available to control type 2 inflammation.

Unknowns and future research highlights in type 2 immune response

- Which cell is more critical and predominant for general type 2 responses, and in which disease?
- Which cytokine is more important for which clinical in vivo situation?
- A detailed list of environmental factors that enhance type 2 responses
- Mechanisms of viral infections in exacerbation of type 2 diseases
- Local immune deficiency caused by a type 2 immune response
- Effect of type 2 immune responses to chronicity
- Novel biomarkers of type 2 responses for treatment selection, to decide when to stop treatment and to monitor therapy response in type 2 diseases
- Role of epithelial barrier leakiness in the development and chronicity of complex type 2 immune response–related diseases
- Head‐to‐head comparison of different type 2 immune response‐targeting treatments
- Pharmacoeconomics of different type 2 immune response‐targeting treatments in comparison with existing conventional treatments
- Disease‐modifying effect of different type 2 immune response‐targeting treatments
- Combination treatments with allergen immunotherapy

#### 2.2.2 T‐regulatory and B‐regulatory cells

Immune regulation is an important function of the immune system to tolerate self‐tissues and non–self‐environmental allergens. T‐regulatory (Treg) cell subsets have distinct phenotypes and include constitutive and inducible subsets of CD4^+^CD25^+ ^Forkhead box P3 (FOXP3)^+^ Treg cells and type 1 Treg cells (Tr1). As a second major player in immune regulation, IL‐10–producing B‐regulatory (Breg) cells have also been demonstrated to suppress allergen‐specific responses and promote IgG4 isotype antibodies.^49^ Allergen tolerance in high‐dose–exposed individuals such as beekeepers and cat owners, the AIT response, and protective effects of farm exposure make up one of the most representative areas where Treg and Breg cells display their major roles.^49^, ^50^, ^51^ IL‐10, IL‐35, and TGF‐beta are the major suppressor cytokines with immune regulatory functions within multiple complex mechanisms.^42^, ^50^, ^52^ Different subsets have been defined in different disease conditions, and research should further identify their regulation and in vivo relevance.^53^ T‐ and B‐regulatory cells suppress many functions of type 2 inflammation including type 2 innate lymphoid cells.^54^ Extensive research is ongoing in this area. To date, there are no biologicals that induce T‐ and B‐regulatory responses in patients; however, various modes of AIT represent major stimulators of these cells in an allergen‐specific manner in vivo.

Unknowns and future research highlights in Treg and Breg response

- Life span of allergen immunotherapy and natural exposure‐induced Treg and Breg cells in vivo
- Effect of Treg and Breg cells on tissue cells
- Functional comparison of different subsets of Treg and Breg cells
- Molecular mechanisms of Treg and Breg cell generation in vivo
- Adjuvants that promote Treg and Breg cells in vivo
- Relationship of resident tissue cells and their interaction with Treg and Breg cells in allergen immunotherapy‐induced immune tolerance
- Early biomarkers and predictors for the generation of Treg and Breg responses
- Mechanisms of long‐term maintenance of allergen tolerance and the link to Treg and Breg responses
- Mechanisms of inducing high‐affinity IgG4 and low‐affinity IgE antibodies

### 2.3 Neuroimmune mechanisms in allergic inflammation

It is becoming increasingly clear that immune cells do not act alone and that cross talk and reciprocal regulation between neural and immune systems are essential in the pathophysiology of allergic diseases including allergic asthma, atopic dermatitis, and food allergies.^55^, ^56^ Immune and neuronal cell types are found in large numbers at skin and mucosal barrier surfaces and are in close contact with each other forming a neuronal‐immune cell network.^57^, ^58^, ^59^, ^60^ Both immune and neural cells detect and respond to environmental threats and harmful stimuli including allergens. Innate and adaptive immune responses mediate proinflammatory responses by secretion of cytokines (eg, IL‐4, IL‐5, IL‐9. IL‐13, IL‐25, IL‐31, IL‐33, TSLP), chemokines (eg, histamine), and other lipid mediators (eg, leukotrienes) on encountering allergens. Within these cytokines, a biological targeting IL‐31 was shown to treat itch in atopic dermatitis. In addition to mediating allergic responses via immune responses, these proinflammatory mediators also directly activate sensory neurons that regulate itch, cough, sneezing, bronchoconstriction, and alterations in gastrointestinal motility.^59^

On stimulation, sensory and autonomic neurons release neuropeptides and neurotransmitters such as substance P, neurokinin A, neuromedin U (NMU), calcitonin gene–related peptide, vasoactive intestinal peptide, acetylcholine, and norepinephrine that signal immune cells.^55^ In the airways, calcitonin gene–related peptide is released by sensory nerves, which has been shown to inhibit dendritic cell maturation and allergen‐specific T‐cell responses.^61^ In the gut, ILC2 cells were shown to express Nmur1, a receptor for the neuropeptide NMU. ILC2s live in close proximity to NMU‐producing nerve cells and become proinflammatory when exposed to NMU. NMU signaling can significantly amplify allergic inflammation when high levels of IL‐25, IL‐33, and TSLP are present.^62^ In a mouse model of allergic asthma, norepinephrine was found to stimulate IgE production on binding β2‐adrenergic receptors and activating B cells.^63^, ^64^ A positive feedback loop between neurotransmitters and neuropeptides and immune cells exists. However, our understanding of these interactions and the signals that mediate their responses to the ever‐changing physiological and pathological conditions is still very limited.

Future prospects for research on neuroimmune regulation of allergic diseases

- The mechanisms underlying allergen‐induced release of proinflammatory mediators and neural activation (reflexes)
- Colocalization and direct and local communications between neuronal and immune cells and their role in mediating allergic response and tolerance
- Identification of neuropeptides and neurotrophins that directly act on immune cells via receptors
- Development of pharmacological compounds targeting neuropeptides and neurotrophins that mediate allergic response
- Feedback loop between neuronal and immune cells in mediating immune homeostasis

### 2.4 Exposome, environmental factors and allergy

The rising trend in allergies is associated with changes in lifestyle and the control of infections which, taken together, seem to result in an “under‐challenged” immune system.^65^, ^66^ On the other hand, lifestyle changes and indoor and outdoor environmental pollutants^67^, ^68^, ^69^ are suspected to keep our immune system in a constant state of low‐grade inflammation. Apart from direct effects of outdoor pollutants on humans, pollen‐producing plants are themselves subject to modification by anthropogenic pollutants.^70^, ^71^ Presumably, beneficial factors include growing up in a rural environment, traditional lifestyle, and a nutrition rich in dietary fibers and of a high diversity. It has become evident that environmental factors induce epigenetic changes which are associated with allergic diseases (summarized in Ref.^72^).

**Figure 2:**
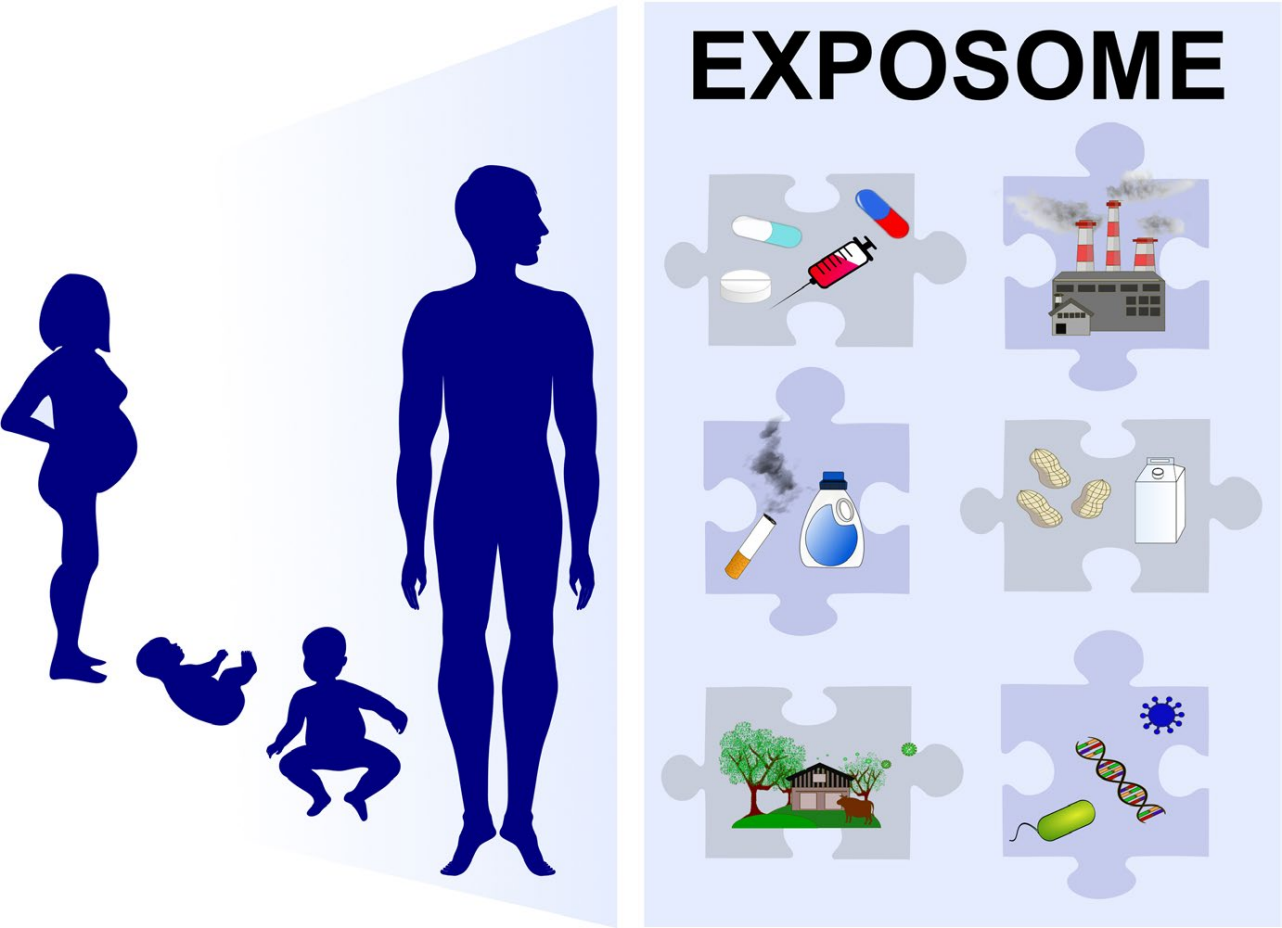
The exposome: The exposome includes the entire environmental exposures of an individual from conception throughout the whole life. Early‐life events such as mode of delivery, breastfeeding, mother's diet, lifestyle and health status, antibiotics, and other drug usage in pregnancy and early childhood, early‐life environment (ie, siblings, pets at home, proximity to farm animals and green areas, usage of primary farm products) can significantly influence the epigenetic regulation of immune system and tissue cells

The exposome includes the entire environmental exposures that a person experiences, from conception throughout the whole life (Figure 2).^73^ A clear missing knowledge is the lack of thorough epidemiological studies encompassing a holistic approach with exposome and reactome (response patterns) over a life span. This wide gap further opens especially because validated methods for exposome assessment, especially the personal one, are lacking. Furthermore, a clear bias emerges since biogenic and anthropogenic pollutants are measured outdoors while people spend a considerable part of their lives indoors. The research focus in environmental health and allergy should therefore be to study the impact of indoor and outdoor pollutants focusing on the role of combined exposures to air pollution, microclimate, green spaces, and allergens. Innovative approaches to characterize environmental exposures including satellite data and stationary and personal monitoring should be developed.^74^ This further requires the development of new informatics tools and data analytics to analyze the large and complex generated datasets. The aim should be to understand the impact of environment through the entire life span on the complete disease spectrum to unravel the interaction of the environment with the barrier organs including their microbiomes, the immune system, and the whole body. Climate change exhibits direct and indirect effects on human health–related aspects. Climate variability modifies the abundance and occurrence of plants and fungi, noticeably those with high allergological importance. The effect of climate change on human health is both a threat and foremost a research focus especially in the field of allergy. A thorough understanding of the molecular mechanisms of the interactions of environment with the human body but also environment‐environment interactions will enable us to develop prevention strategies for allergies.

Hot spots in environmental health research

- Moving from associations to causalities and molecular mechanisms
- Understanding environment‐gene interactions and especially the role of epigenetic changes
- Develop innovative methods for exposome assessment, especially the personal one
- Development of devices for personal monitoring of real‐time pollen and fungal spore abundance spatiotemporal information
- Understanding additive and summative effects of environmental factors on health and disease
- Define personal thresholds for environmental triggers for allergic symptoms

### 2.5 Role of the microbiome and diet in immune responses

Enormous varieties of microbes colonize the skin and mucosal body surfaces. These microbes are organized within complex community structures, whose composition is dependent on the specific body site examined. It is increasingly appreciated that the microbiome interacts intimately with mucosal immune processes and disrupted communication between the microbiome and the host due to altered microbiome composition and/or metabolism is thought to negatively influence immune homeostatic networks and may play a role in immune hypersensitivity to environmental exposures, such as allergens.^75^

A number of studies have consistently demonstrated that an altered gut, lung, nasal, or skin microbiome is associated with, and sometimes precedes, allergic sensitization and inflammation.^76^, ^77^ In particular, early‐life events such as mode of delivery, breastfeeding, mother's diet and health status, antibiotics, and other drug usage in pregnancy and early childhood, early‐life environment (ie, siblings, pets at home, proximity to farm animals and green areas, usage of primary farm products) can significantly influence the timing of bacterial colonization and establishment.^78^, ^79^, ^80^ However, one of the most potent modulators of microbiota composition is diet, as consumed foods provide the fuel for microbial metabolic activities.^81^ For example, microbiota‐accessible carbohydrates (MAC) are complex carbohydrates found in dietary fibers that contribute to microbial diversity and generation of metabolites, such as short‐chain fatty acids (SCFAs).^82^ SCFAs promote regulatory immune responses, and high SCFA levels early in life are associated with protection from atopic sensitization.^83^ In contrast, a high‐fat diet is associated with negative effects on microbiota composition and metabolism.

Despite increasing awareness of the importance of microbiome‐diet interactions in health and disease, the molecular basis for these multidirectional functional interactions is only beginning to be described. Although diet‐microbiome interventions are exciting and attractive approaches, many unknown factors still limit the successful translation of these potentially game‐changing interventions into the clinic.

Unknowns in the microbiome area for future research

- Contribution of the virome (viral communities) and mycobiome (fungal communities) to immune tolerance networks
- Mechanistic pathways linking diet diversity, microbial metabolism, and allergy prevention
- Role of the diet in modulating microbial communities outside of the gut
- The importance of baseline microbial populations or historical dietary patterns in determining the response to microbiota or diet‐based interventions
- The influence of microbiota on the clinical response to allergen‐specific immunotherapy and its mechanisms
- Definition of a healthy microbiota and ways to achieve it
- Possibility of intervention in the microbiome of a diseased individual

### 2.6 Respiratory viral infections and allergy

Respiratory viruses are the most common causes of respiratory diseases, which can be linked with the potentiation of acute and chronic respiratory mucosal inflammation. This usually occurs through mechanisms including upregulation of cell adhesion molecules, pathogen sensing receptors, and Toll‐like receptors, which are common immunopathogenic factors mediating or involved in virus‐ and allergen‐induced mucosal inflammation.^84^, ^85^, ^86^, ^87^, ^88^, ^89^, ^90^, ^91^ In addition, respiratory viruses were suggested to impact cilia and tight junction integrity in airway epithelial cells through the modulation of ZO‐1, claudin‐1, and occludin in the airway epithelial barrier,^92^, ^93^, ^94^ which may be linked to pathophysiology of airway diseases. The nasal epithelium is the primary portal of entry for respiratory viruses and immediate target for viral replication in the airways.^87^, ^95^ It is also an active component of initial host responses against viral infection. Such nasal epithelial‐specific transcriptomic alterations may significantly influence the downstream immune responses and homeostasis that define the pathology of respiratory infection and complications.^87^, ^95^, ^96^, ^97^ This is evident in the case of most respiratory viral infections, which, while self‐limiting, could trigger chronic type 2 inflammatory responses via excessive release of chemokines and cytokines into the airways. The resulting recruitment of the immune cells (ie, neutrophils, eosinophils, mast cells, and T cells) may then ultimately predispose the airway to remodeling.^98^, ^99^, ^100^, ^101^ In addition, a recent study showed that H3N2 infection of the nasal epithelium was associated with significant increase in interferons (IFN‐α, IFN‐γ, IL‐29), proinflammatory cytokines (TNF‐α, BDNF, IL‐3), and viral‐associated chemokines (IP‐10, MCP‐3, I‐TAC, MIG), detectable as early as 24 hours postinfection.^102^ This translates into rapid monocyte, NK‐cell, and innate T‐cell (MAIT and γδ T cells) activation, evident with CD38+ and/or CD69+ upregulation.^102^ Therefore, an understanding of the predominant type and underlying mechanisms of mucosal inflammation triggered by common viral infections will allow identification of targets for better management of chronic airway inflammatory diseases.

Unknowns and future prospects for research in viral infections and allergic diseases

- The predominant type and underlying mechanisms of mucosal inflammation (eg, type 2 or non‐type 2) triggered by infection of different types of respiratory viruses
- Mechanistic role of viral infections in chronicity
- Mechanisms of viral infections in exacerbations
- Mechanisms of viral infections in breaking of allergen tolerance
- Novel mechanisms to prevent or avoid viral infections
- Novel vaccines for various viruses
- Novel anti‐viral treatments based on newly identified mechanisms

## 3 DIAGNOSTIC CHALLENGES AND REGULATORY CONSIDERATIONS

### 3.1 Clinical trials for the treatment of allergic diseases

In 2008, the Committee for Medicinal Products for Human Use (CMPH) of the European Medicine Agency (EMA) has implemented the “Guideline on the Clinical Development of Products for Specific Immunotherapy for the Treatment of Allergic Diseases (CHMP/EWP/18504/2006)” (Available from: http://www.ema.europa.eu/docs/en_GB/document_library/Scientific_guideline/2009/09/WC500003605.pdf and ref.^103^) and by this has set methodological standards for clinical trial designs for AIT regarding phase I‐III performances and outcomes. This guidance has been followed in “Therapy Allergen Ordinance (TAO)” which has been initiated for future registration and marketing authorization in Germany of a group of allergen extracts of specific species (details in Ref.^104^). An increasing number of AIT products fulfill the regulatory demands and have been authorized in different countries on the basis of proven efficacy in the clinical documentation.^105^, ^106^ However, the regulatory guidance leaves some space for interpretation regarding certain specificities of clinical trial design in both early and late phases of clinical development programs for AIT, and harmonization of methodological principles in the design of these trials would be preferable for all parties involved.^107^ Hence, the Immunotherapy Interest Group (IT‐IG) of the European Academy of Allergy and Clinical Immunology (EAACI) has elaborated different task‐force projects regarding improvement of methodological study design in AIT (https://www.eaaci.org/organisation/eaaci-interest-groups/ig-on-immunotherapy/activities/2880-task-forces-of-the-immunotherapy-interest-group.html; accessed on 07 Dec 2018). In an EAACI Position Paper, this group has aimed to summarize and standardize clinical endpoint measures and has elaborated a combined symptom and medication score (CSMS) as standard primary endpoint for future (pivotal) trials in AIT.^108^ Another example is an EAACI Position Paper, which overviews current concepts in tolerance‐inducing mechanisms aimed to highlight potential biomarkers which may be of predictive value for determining responders to AIT in clinical trials.^15^ However, there is an urgent need for further harmonization and clinical validation of methodological determinants in AIT clinical study design, which can only be achieved by international collaboration of clinical experts, methodologists, and regulatory authorities.^109^, ^110^

Examples of unknowns and future prospects for harmonization of AIT trial design and interpretation of trial results (modified to references^107^, ^109^, ^110^)

- Evaluation and validation of possible biomarkers of predictive value for efficacy^15^
- Further validation of clinical meaningful primary and secondary endpoints^108^
- Clinically justified definitions of relevant treatment effect sizes
- Potential of allergen exposure chambers for AIT product development^107^
- Minimal level of evidence needed for the clinical documentation of efficacy in the pediatric population^110^
- Better understanding of placebo effects in SCIT and SLIT^107^
- Defining clinical endpoints and establishing the effectiveness, disease‐modifying properties, and the duration of both in asthmatic patients

### 3.2 Precision medicine and immune monitoring of allergic diseases

**Figure 3:**
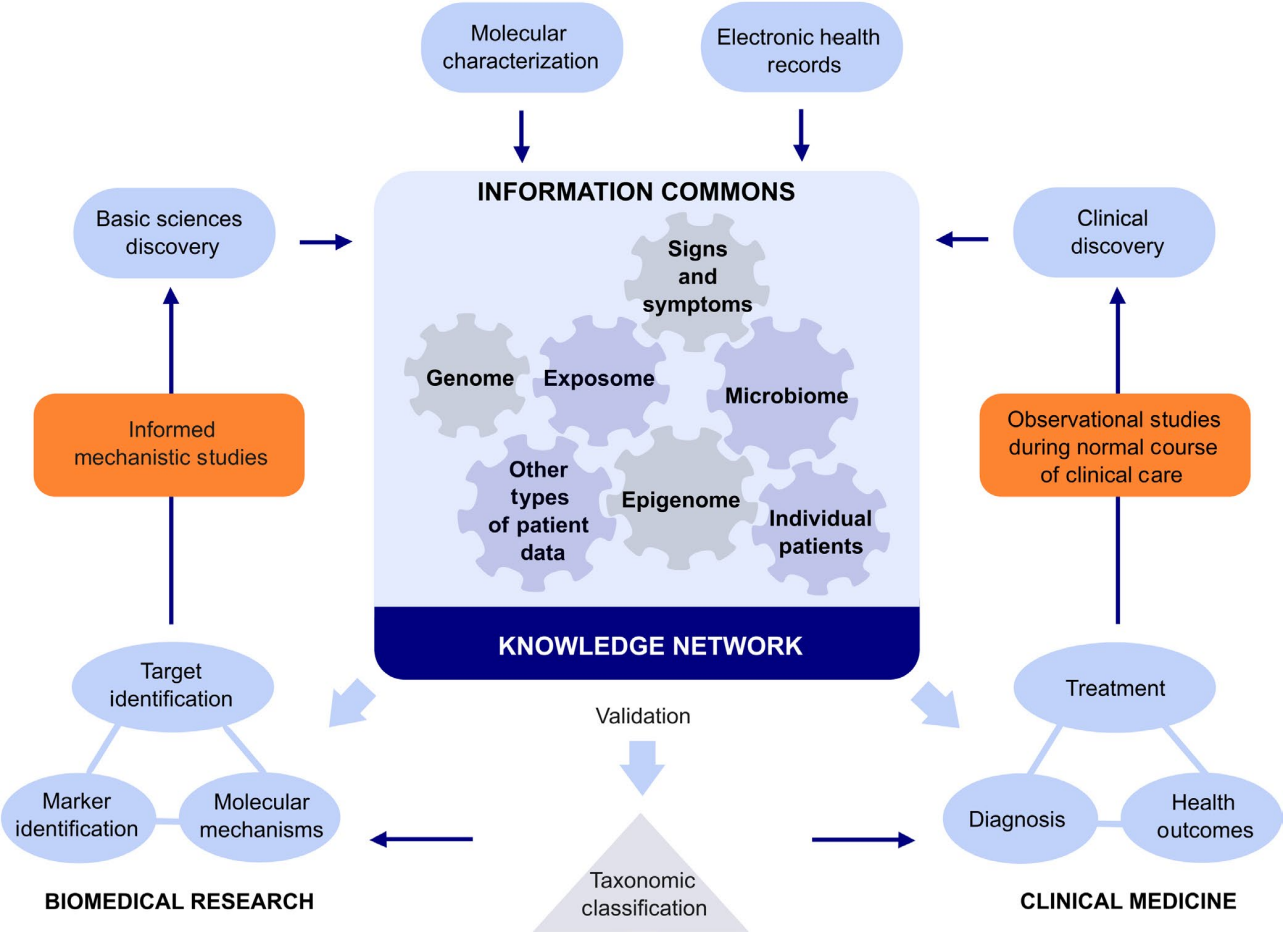
Precision medicine: Precision medicine requires the network of knowledge from both biomedical and clinical research. It includes all of the omics areas and exposome from molecular characterization and biomarker development to electronic health records, and clinical discoveries in diagnosis and treatment. The introduction of a new taxonomy is needed to ensure that all the stakeholders speak the same language

**Figure 4:**
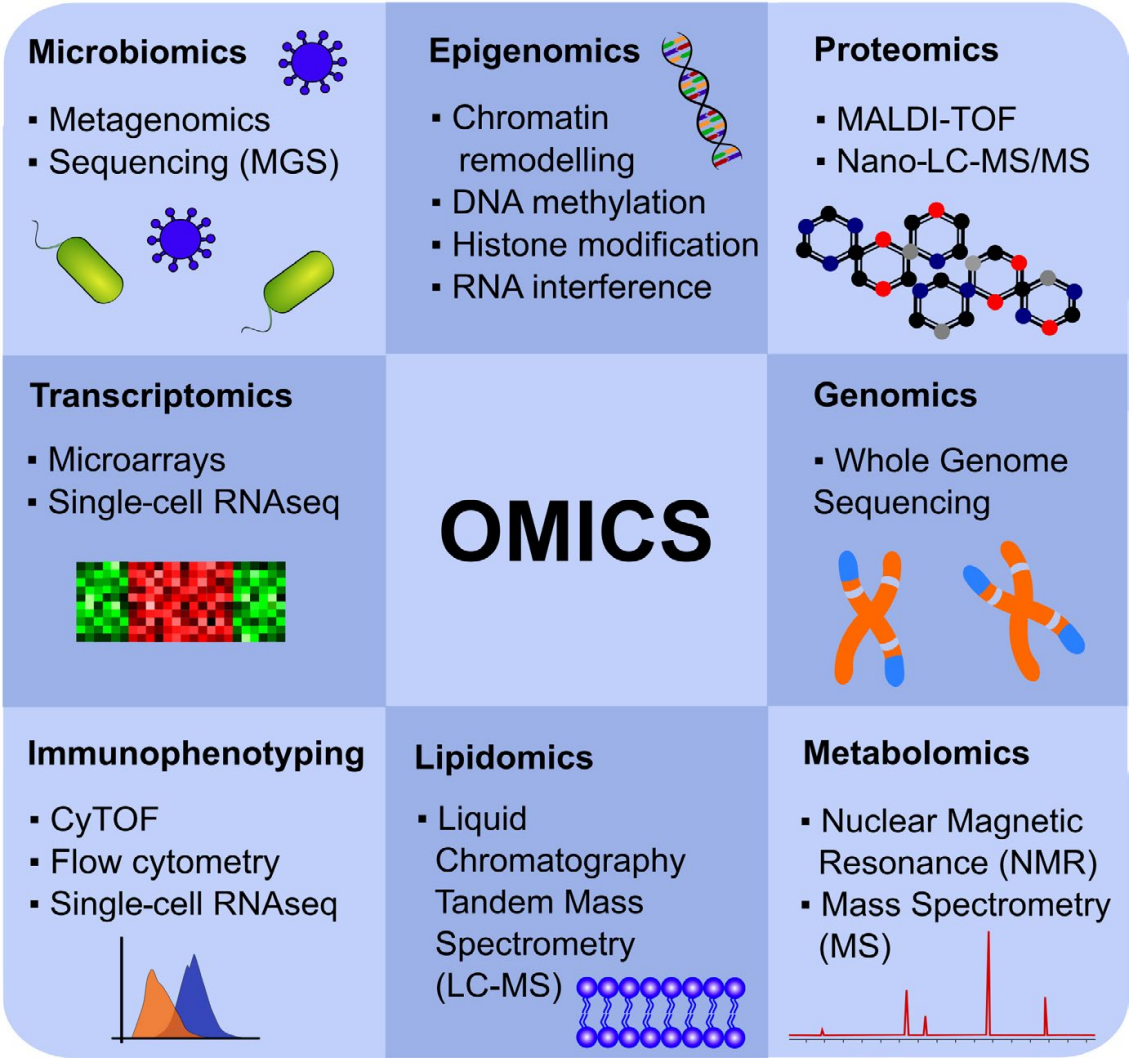
Omics: The omics revolution was one of the major driving forces of recent developments that enabled investigation of almost everything at the molecular level of proteins, lipids, and small molecules including innumerable DNA and RNA sequencings with a hypothesis‐free approach

Precision medicine (providing the right treatment to the right patient and the right dose at the right time) requires an accurate diagnosis and monitoring of the treatment response. While precision medicine has been practiced in allergology for over a century since the advent of grass pollen‐specific immunotherapy,^111^ it currently infers (often synonymous with “personalized medicine”) use of the new “omics” technologies to identify genes or biomarkers for diagnosis or monitoring of treatment efficacy (Figure 3).^13^, ^112^ The “omics” revolution is based on platform technologies in genomics (by far the most robust), metabolomics, proteomics, epigenomics, transcriptomics, lipidomics, and microbiomics to generate vast global datasets, and advanced bioinformatics to interrogate and interpret the datasets using machine learning and artificial intelligence (Figure 4).^113^ Such analysis of population‐based datasets can reveal novel insights to underpin therapeutic selection from an expanded range of precise biologicals.^114^ Examples are emerging from patients with inborn errors of immunity (IEI) in whom the genetically defined defect can be specifically targeted with therapeutics.^115^, ^116^ The functional utility of data from the omics platforms will be further enhanced by the public release of omics datasets including Genotype‐Tissue Expression (GTEx)^117^ and Encyclopaedia of DNA Elements (ENCODE).^118^

Technological advances in immune monitoring capability are augmented by highly standardized or chimeric recombinant allergens and peptides (B‐ and T‐cell epitope‐based). Exponential advances in microarrays, time‐of‐flight mass cytometry (CyTOF), basophil activation tests, next‐generation gene sequencing, and RNA‐seq are generating huge enabling datasets.^15^, ^115^, ^116^, ^119^, ^120^, ^121^, ^122^, ^123^ The risk that small populations of highly pathogenic cells might be masked by abundant signatures of more frequent or more active cells is combated by the emergence of enhanced single B‐ and T‐cell immunophenotyping using flow cytometry–based assays. This allows longitudinal immunoprofiling of relevant cell subsets in individuals before and during AIT. Better dissection of mechanisms underlying allergic diseases informs better tailoring of therapies.^115^, ^116^, ^119^, ^120^, ^121^, ^122^, ^124^, ^125^

Unmet needs in precision medicine

- Consensus on endotypes and clinically applicable biomarkers for distinct allergic disorders
- Accurate targeted activity; even, widespread or specific as needed
- Increased availability of oral formulations: solid or liquid
- More favorable dosing intervals
- Minimization of adverse side effects (especially anaphylactic/allergic reactions, antibody induction, complement activation)
- Economic feasibility enhancement
- Determination of long‐term effects
- “Large dataset” protection and interpretation, including ethical considerations
- Development of a precision medicine‐specific nomenclature
- Development of treatment and diagnostic algorithms
- Development of precision medicine–focused guidelines

### 3.3 Allergic rhinitis and nonallergic rhinitis

Chronic rhinitis (CR) is one of the most common diseases globally, with a considerable financial burden.^126^, ^127^ At present, CR is simplistically subclassified as allergic rhinitis (AR) and nonallergic rhinitis (NAR).^128^ Although phenotyping of rhinitis has important consequences in the treatment of the entity,^129^ the presently employed phenotypes cannot meet the needs of precision medicine; suggesting an urgent need for the CR phenotypes to be updated with the progress of diagnostic methods. In this regard, Meng and colleagues have recently investigated the phenotypes of CR based on a cluster analysis of 12 clinical variables.^130^ In this study, AR was subclassified as allergic rhinitis with or without asthma, while NAR was subclassified as nonallergic rhinitis with eosinophilia syndrome (NARES) without asthma, NARES with asthma, local allergic rhinitis (LAR), and idiopathic rhinitis. The finding of the LAR cluster was interesting because none of these patients had a history of asthma, but demonstrated high levels of local eosinophils and local production of specific IgE (local IgE), of which the latter has been used in studies of LAR over the last few years.^131^, ^132^, ^133^ Indeed, another study by Meng and colleagues has suggested that local IgE is a reliable noninvasive alternative to serum IgE for the diagnosis of AR,^134^ and there is emerging evidence that local IgE could also be used instead of nasal allergen provocation test (NAPT) for the diagnosis of LAR. Besides local IgE, nasal cytology has also been shown to be useful in the diagnosis of CR A recent study by She and colleagues assessed nasal cytology in chronic sinusitis patients with rhinitis, using the liquid‐based ThinPrep Cytology Test (TCT) and demonstrated that this technique has higher sensitivity, specificity, and positive predictive value for inflammation in the inferior turbinates than for inflammation in the maxillary sinus.^135^ Thus, the TCT might also be used in the studies on CR, especially NARES.

In conclusion, there is increasing evidence that local IgE and nasal cytology are useful clinical diagnostic markers in CR and might represent the way forward especially for studies differentiating the endotypes of AR and NAR in the future.

Future research questions and prospects for chronic rhinosinusitis

- Could local IgE measurement be used for the diagnosis of local allergic rhinitis?
- Could nasal cytology be used in the study of chronic rhinitis, especially for the differential diagnosis of chronic rhinitis?
- Can we provide the diagnostic standard of local IgE determination for allergic rhinitis and local allergic rhinitis?
- Can we provide the diagnostic standard of nasal eosinophil count for nonallergic rhinitis with eosinophilia syndrome?

### 3.4 New approaches to the diagnosis and treatment of drug hypersensitivity reactions

Drug hypersensitivity reactions (DHRs) are defined as adverse effects of pharmaceutical formulations that clinically resemble allergy. Drug allergies are defined as DHRs for which a definite immunological mechanism, IgE‐ or T cell–mediated, is demonstrated.^136^ DHRs constitute an important health problem, affecting more than 7% of the population, 137 for whom drugs, such as beta‐lactam antibiotics and nonsteroidal anti‐inflammatory drugs, are essential for treatment of common diseases.^138^, ^139^ Based on the clinical characteristics of DHRs, different phenotypes have been identified,^137^ although the lack in understanding the underlying mechanisms of many DHRs has hampered the definition of endotypes and identification of biomarkers.^140^, ^141^

The classification of DHRs based on the time elapsed between drug administration and development of symptoms is still a matter of debate, because it is difficult to establish a cutoff point to distinguish between immediate and nonimmediate DHRs.^13^, ^136^, ^141^ These data are relevant for defining phenotypes and establishing an accurate diagnosis and specific treatment. An important recent advance has been the inclusion of “Drug hypersensitivity” as a subsection in the International Classification of Diseases (ICD)‐11.^140^

The diagnosis of DHR is mainly based on skin tests and drug provocation tests, methods that are not free of risk, still lack standardization, and differ depending on the drug, mechanisms, and even the health system.^142^, ^143^, ^144^ There is an urgent need for developing new in vitro diagnostic tests or improving those already existing,^138^ such as basophil activation test,^145^, ^146^ to improve the diagnostic workup. The complexity of DHR diagnosis and its lack of optimal specificity lead to an over‐diagnosis. This is an important problem, as patients “labeled” as allergic receive alternative treatments that are usually less effective and more toxic, so “de‐labeling” constitutes a public health measure.^147^ Primary care physicians are often the first point of contact for patients with DHRs; thus, they have a key role in diagnosis and need specific training.^148^

Although the specific treatment of DHRs is avoiding the drug involved and those chemically related, desensitization is nowadays a frequent option.^149^, ^150^, ^151^ In that sense, rapid drug desensitization is a cost‐effective technique that activates inhibitory mechanisms and permits patients to receive the first‐choice medications to which they are allergic.^149^, ^150^

Research needs for DHRs

- The prevalence and incidence of DHRs
- Underlying mechanisms of DHRs
- The most adequate classification of DHRs
- Definition of endotypes and biomarker identification
- The most accurate diagnostic approach:
  i Skin test standardization, sensitivity, specificity, and predictive values for most drugs
  ii A standardized protocol for performing drug provocation test
  iii The role of in vitro tests for diagnosis
- The mechanism of inhibition in rapid drug desensitization procedure

## 4 UNMET NEEDS AND FUTURE RESEARCH AREAS IN TREATMENT OF ALLERGIC DISEASES

### 4.1 How to treat food allergy in the future: new developments and concepts

We are observing a pandemic increase in food allergy and approaching an era of efficient treatments. In peanut allergy oral immunotherapy (OIT), phase III studies on AR101^152^ in peanut allergic patients and phase II(b) and III studies on epicutaneous immunotherapy (EPIT) for milk and peanut^153^, ^154^ have been conducted. The FDA application for AR101 is submitted, while peanut EPIT submission has been retracted to provide additional technical information. Different ways of application differ with regard to efficacy of desensitization; however, all current applications are linked to an avoidance regimen and it is unclear how long the individual treatment needs to be applied. Consistent data from conventional high‐dose milk, peanut, and egg OIT report good efficacy with regard to desensitization.^155^, ^156^ Therefore, in addition to these highly standardized products, OIT using conventional food sources may become a more frequent treatment offered by clinicians in the community as a result of excessive demand in the absence of guidelines and recommendations. As a first step, the European Academy of Allergy and Clinical Immunology (EAACI) stated to consider OIT for these three foods in settings with the appropriate infrastructure and experience.^155^

The major issues in treating food allergy by immunotherapy are safety, the low rate of tolerance induction,^155^ a high rate of side effects and dropouts,^153^ a lack of understanding of the optimal dose and time of treatment, and the existence of only few, suboptimal biomarkers that predict treatment response and how to perform multifood OIT.^13^, ^157^, ^158^ These limitations are addressed in numerous treatment approaches: (a) peptide immunotherapy targeting the T‐cell compartment and lacking IgE cross‐linking^159^, ^160^, ^161^; (b) hypoallergenic variants of allergens or extracts by chemical or thermal modification^162^, ^163^ or mutations which combine reduced desensitization with a minimally altered T‐cell epitope diversity^164^; (c) the usage of immunomodulatory substances and/or particles^165^; (d) the addition of prebiotics and/or probiotics^76^, ^166^; (e) the application of biologics either alone^167^, ^168^ or as adjuvants of OIT^169^, ^170^; (g) very low dose OIT^171^, ^172^; and (h) sublingual OIT.

Recent methodological developments on cloning and antibody generation from single‐cell sorting of allergen‐specific B cells will allow novel insights on the nature of peanut‐specific B‐cell responses and may give rise to novel high‐affinity blocking antibody treatments.^173^

Unknowns in the treatment of food allergy

- Which markers predict treatment response?
- Which markers can be used to monitor tolerance development?
- What is the optimal dose and time of treatment?
- Is there a role for biologics to improve safety and efficacy of immunotherapeutic approaches?
- What is the best route to apply immunotherapy?
- How can we implement oral immunotherapy safely in a community setting?
- How to modify allergen formulations for tolerance induction?

### 4.2 Treatment of allergic diseases with biologics

Molecular mechanisms of type 2 inflammation in allergic disease are discussed above. Treatment of allergic disease with biologicals particularly targets type 2 inflammation. For several years, omalizumab was the only broadly applied biological in allergic diseases in childhood and adult asthma^174^ and chronic urticaria.^8^, ^175^ Recently, phase III trials demonstrated efficacy by blocking the IL‐4/IL‐13 pathway in glucocorticoid‐dependent severe asthma, moderate‐to‐severe uncontrolled asthma,^176^, ^177^ CRSwNP,^178^ and atopic dermatitis^179^ (dupilumab), by blocking IL‐5 in severe eosinophilic asthma (mepolizumab,^180^ reslizumab^181^) and CRSwNP and severe uncontrolled asthma by blocking the IL‐5 receptor (benralizumab^182^, ^183^).^184^ FDA and EMA approved mepolizumab, reslizumab, and benralizumab for adult uncontrolled asthma and dupilumab for atopic dermatitis in adolescents and adults, and these biologics are integrated in current guidelines and position papers.^12^, ^175^, ^185^, ^186^ New indications for these biologicals can be expected in the near future.^184^, ^187^

Novel data arising from a long pipeline of cytokine and chemokine receptor targeting drugs will lead to additional treatment options and change the landscape of therapeutics in other atopic diseases including food allergy, chronic rhinosinusitis with nasal polyps,^12^, ^188^ and systemic mastocytosis.^189^ Biologics may also increase efficacy and safety of AIT.

Phase II trials of biologics targeting type‐2 pathways beyond IL‐4, IL‐5, and IL‐13 are encouraging. Tezepelumab blocking the TSLP receptor showed efficacy in uncontrolled asthma independent of eosinophil counts.^190^ Nemolizumab blocks the IL‐31R‐alpha and reduces pruritus and to a certain extent also dermatitis severity.^191^ It is a good example for biologicals with the potential to be combined with a second to achieve better disease control. Another important group of emerging biologics will address mucosal inflammation^44^, ^192^ and upstream events which are key for innate lymphoid cells such as anti–IL‐25 and anti–IL‐33.

Costs are an important factor when prescribing biologics. Currently, direct treatment expenses only partially contribute to the overall disease‐associated financial burden.^193^, ^194^ Thus, costs related to comorbidities^195^ and the impact of biologics on these factors will be key. The development of biomarkers, prediction models,^5^, ^196^ the design of trials comparing different biologics and the implementation of strategies to investigate the safety, function, and efficacy in children, the elderly, and pregnant women represent additional crucial challenges that need to be answered in the near future.

Gaps in the treatment of allergic disease with biologics

- How to predict treatment response?
- Will new biologics help to promote tolerance induction?
- How to define precision medicine approaches to treat severe and complex atopic phenotypes?
- Long‐term side effects of biologics?
- Safety and efficacy of biologics in childhood, in pregnancy, and in elderly?
- Novel biomarkers and sets of biomarkers will be needed.
- Treatment algorithms and guidelines for biologics usage are needed.

### 4.3 Small molecules for the treatment of allergic asthma

Several targeted therapeutic options for asthma and related conditions have been licensed in the past two decades. Apart from parenteral monoclonal antibodies directed against key inflammatory targets, small molecules comprise another class of systemic medication interfering with inflammatory pathways underlying these disorders.^197^ Leukotriene modifiers (LM), and specifically cysteinyl leukotriene (CysLT) receptor 1 antagonists (LTRA), are the first small molecule agents widely applied for targeted treatment of asthma and comorbid AR both in adults and in children.^198^ Being launched in an evolving era and lacking adequate biomarkers, initial positioning of anti‐leukotrienes in asthma treatment has been mainly based on their efficacy in clinical models and not on adequate patient stratification which may have delayed proper positioning of this targeted therapy.^199^

More recently, another class of lipid mediator antagonists entered clinical development: antagonists of the prostaglandin D2 (PGD2) receptor DP2 also known as *chemoattractant* receptor‐*homologous* molecule expressed on Th2 cells (CRTH2).^200^ DP2/CRTH2 receptors are present on several inflammatory cells including mast cells, T‐helper 2 cells, type 2 ILCs, and eosinophils, and hence, PGD2 plays an important role in linking both the innate and adaptive immune system through type 2 responses.^201^ Although two compounds showed (modest) efficacy in allergen challenge,^202^, ^203^ many CRTH2 antagonists failed in later clinical phases, possibly due to inadequate (non‐type 2) patient populations. With the emerging evidence of an upregulated PGD2 pathway and its association with type 2 inflammation in uncontrolled severe eosinophilic asthma,^204^ more recently, several CRTH2 antagonists have been tested in type 2 conditions, including allergic and/or refractory eosinophilic asthma, showing improvements in several clinical outcomes.^205^, ^206^, ^207^, ^208^, ^209^ In a post hoc analysis, CRTH2 antagonist OC000459 (Timapiprant) appeared most effective in younger (≤40 years) patients with uncontrolled atopic eosinophilic asthma (blood eosinophils ≥ 250 cells/μL).^209^ Currently, several CRTH2 antagonist programs are in phase III studies which should help to consolidate phenotypes and biomarkers responding to these targeted drugs. Additionally, while the same immune/inflammatory cells express both CysLT1 and DP2/CRTH2 receptors, further research is warranted on potential synergistic effects of LTRA and CRTH2 antagonists in T2 inflammatory conditions.

Research needs for treatment with novel small molecules

- Sensitive and reliable point‐of‐care biomarkers to identify potential responders and to monitor (long‐term) effects of anti‐lipid mediator small molecules (LM, LTRA, CRTH2 antagonists).
- Combining LTRA and CRTH2 antagonists may be beneficial in patients with type 2 inflammatory conditions and, hence, warrants further clinical investigation.
- Since both DP2/CRTH2 receptors and CysLT1 receptors are present on both immune/inflammatory and structural cells, apart from anti‐inflammatory activity, blocking these receptors may potentially have disease‐modifying effects (“anti‐remodeling”).

### 4.4 Novel therapeutic concepts in AIT for airway disease

Allergen‐specific immunotherapy not only reduces symptoms in patients with AR,^106^ LAR,^1^ and asthma,^106^, ^210^ but there is also evidence that AIT can reduce the development of asthma and new sensitizations,^210^, ^211^, ^212^ thus being the only available disease‐modifying treatment. Altogether, albeit not all of the highest quality, there is evidence that AIT can halt the allergic march in patients with AR.^213^ Moreover, there is some evidence that AIT is cost‐effective in AR with or without asthma.^214^

Allergen‐specific immunotherapy is usually given as subcutaneous injections^215^, ^216^ or sublingually,^217^, ^218^, ^219^ but novel treatment forms such as peptide immunotherapy,^11^, ^220^ intralymphatic immunotherapy^11^, ^220^ and use of recombinant allergens, and immune‐modulating adjuvants and nanoparticles^21^, ^220^, ^221^ are under development.

Despite this positive profile, AIT is only used for highly selected patient groups in most countries in Europe.^109^ The reasons for this limited penetration are multifold^16^, ^103^, ^222^ but the long duration of the treatment, the potential side effects especially in groups that could most benefit from AIT, and the inability to predict development of allergic disease and response to AIT treatment are among the most important ones. Recently, EAACI has been very active in providing guidelines for immunotherapy^109^, ^223^ to help physicians and patients in their decisions. However, for further expansion of AIT, we must influence the balance between allergenicity and immunogenicity, which can improve both duration of treatment and create a better side effect profile. Furthermore, we need greater understanding of the molecular mechanisms underlying the development of respiratory allergic disease and of AIT at the level of the individual patient, facilitating better patient stratification for AIT to further improve optimal personalized treatment.^16^, ^107^

Research needs for novel AIT vaccines

- Short, effective, and safe treatment
- Prediction of individual success before or early during the treatment
- Better understanding of the underlying molecular and immunological mechanisms
- Optimal AIT clinical trials that reduce bias and heterogeneity
- Collaboration between physicians, patient organizations, companies, and regulators

## 5 CONCLUSIONS

Our specialty has been evolving at full speed with the introduction of several novel concepts such as knowledge of structures and biological functions of allergens to better understand what makes them allergenic, molecular mechanisms of the type 2 immune response and immune tolerance. Due to the omics revolution and harnessing artificial intelligence to handle huge global datasets to facilitate accurate diagnoses and precise and personalized monitoring of disease, novel treatments are highly expected to further evolve. Like many other disciplines, we are experiencing the early days of the development of new biologicals that have entered the clinic. Small molecules and combinations may offer a rational alternative for the treatment of specific subtypes of asthma and related diseases. Future studies and head‐to‐head comparisons with the more expensive biologics should provide the answer. AIT is the only disease‐modifying treatment option for allergic patients. Despite its overall favorable profile, the use of AIT in many countries is still limited. For further dissemination of AIT, we must influence the balance between allergenicity and immunogenicity, improve the vaccines with the hope for long‐term cure in many patients, and develop novel prevention modalities for early intervention, which can overall improve the efficacy of treatment and create a better side effect profile. As shown in the text boxes in each of the sections, still many questions are waiting to be answered.

## CONFLICTS OF INTEREST

Author ZD reports personal fees from Aquilon, ALK, AstraZeneca, Boehringer Ingelheim, Gilead Hal Allergy, MSD, and Sanofi‐Genzyme, during the conduct of the study. Apart from academic affiliations, ZD works at a phase I/II unit performing clinical studies for different biotech and pharma companies. Author TE reports other from DBV, grants from the Innovation fund Denmark, outside the submitted work; TE is the Co‐I or scientific lead in three investigator‐initiated oral immunotherapy trials supported by the Allergy and Anaphylaxis Program Sickkids. Author KN reports personal fees from Regeneron, grants from NIAID, FARE, and EAT, outside the submitted work; other from Novartis, Sanofi, Astellas, Nestle, BeforeBrands, Alladapt, ForTra, Genentech, AImmune Therapeutics, and DBV Technologies, outside the submitted work. Author REO’H reports other potential financial activities from Aravax Pty Ltd and Paranta Bio Pty Ltd, outside the submitted work. Author OP reports personal fees from Novartis Pharma, MEDA Pharma, Mobile Chamber Experts (a GA2LEN Partner), Pohl‐Boskamp, Indoor Biotechnologies, and Astellas Pharma Global, outside the submitted work; grants and personal fees from ALK‐Abelló, Allergopharma, Stallergenes Greer, HAL Allergy Holding B.V./HAL Allergie GmbH, Bencard Allergie GmbH/Allergy Therapeutics, Lofarma, ASIT Biotech Tools S.A., Laboratorios LETI/LETI Pharma, and Anergis S.A., outside the submitted work; grants from Biomay, Nuvo, Circassia, and Glaxo Smith Kline, outside the submitted work. Author CAA reports grants from Allergopharma, Idorsia, Scibase, Swiss National Science Foundation, Christine Kühne‐Center for Allergy Research and Education, European Commission’s Horison's 2020 Framework Programme, Cure, advisory board of Sanofi‐Aventis/Regeneron, grants from Novartis Research Institutes, and Astra Zeneca, outside the submitted work. Authors HB, WF, LO’M, MJT, CTH, DYW, and LZ report no conflicts of interest in relation to this work.
